# A Study on the Application of the Information-Motivation-Behavioral Skills (IMB) Model on Rational Drug Use Behavior among Second-Level Hospital Outpatients in Anhui, China

**DOI:** 10.1371/journal.pone.0135782

**Published:** 2015-08-14

**Authors:** Cheng Bian, Shuman Xu, Heng Wang, Niannian Li, Jingya Wu, Yunwu Zhao, Peng Li, Hua Lu

**Affiliations:** 1 The First Affiliated Hospital of Anhui Medical University, Hefei, Anhui, PR China; 2 Guangdong Provincial Maternal and Child Health Hospital, Guangzhou, Guangdong, PR China; 3 The Fourth Affiliated Hospital of Anhui Medical University, Hefei, Anhui, PR China; 4 Anhui Provincial Hospital, Hefei, Anhui, PR China; University of Brescia, ITALY

## Abstract

**Background:**

The high prevalence of risky irrational drug use behaviors mean that outpatients face high risks of drug resistance and even death. This study represents the first application of the Information-Motivation-Behavioral Skills (IMB) model on rational drug use behavior among second-level hospital outpatients from three prefecture-level cities in Anhui, China. Using the IMB model, our study examined predictors of rational drug use behavior and determined the associations between the model constructs.

**Methods:**

This study was conducted with a sample of 1,214 outpatients aged 18 years and older in Anhui second-level hospitals and applied the structural equation model (SEM) to test predictive relations among the IMB model variables related to rational drug use behavior.

**Results:**

Age, information and motivation had significant direct effects on rational drug use behavior. Behavioral skills as an intermediate variable also significantly predicted more rational drug use behavior. Female gender, higher educational level, more information and more motivation predicted more behavioral skills. In addition, there were significant indirect impacts on rational drug use behavior mediated through behavioral skills.

**Conclusions:**

The IMB-based model explained the relationships between the constructs and rational drug use behavior of outpatients in detail, and it suggests that future interventions among second-level hospital outpatients should consider demographic characteristics and should focus on improving motivation and behavioral skills in addition to the publicity of knowledge.

## Introduction

At the 1985 conference of the World Health Organization (WHO) in Nairobi, the WHO defined rational drug use (prescription drug use and not street or illegal drug use) in the following terms: “The rational use of drugs requires that the patient receive medications appropriate to their clinical needs, in doses that meet their own individual requirements for an adequate period of time, and at the lowest cost to them and their community”. [1] In our country, *The Core Information Interpretation of Health Education in Rational Drug Use*, which was released by the State Health and Family Planning Commission of the People’s Republic of China on December 10, 2013, defined rational drug use as follows: “Rational drug use is referring to safe, effective and economical drug use. The priority of using essential drugs is one of the most important measures for the rational use of drugs. Irrational drug use will affect health and even be life-threatening”. [2] According to the WHO report, nearly half of global medication use is irrational. Therefore, patients are suffering from drug resistance and even death [3]. The phenomenon of irrational drug use is particularly acute in China, where irrational drug use behavior is observed in 12% to 32% of all patients [4].

To ensure safe medication, China has attempted many strategies, including the introduction of the rational use of drugs platform in the network information environment, the adoption and release of policies and regulations related to the rational use of drugs, and the strengthening of the supervision of rational drug use in hospitals and community pharmacies. Many domestic researchers have conducted studies on the rational use of drugs. However, the research with regard to residents’ rational drug use was only limited to their beliefs and perceptions of rational drug use [5–7]. Thus far, there remains a lack of a complete theoretical framework to assess as to the factors that drive rational drug use behavior.

The Information-Motivation-Behavioral Skills (IMB) model is a theoretical framework developed by Fisher and colleagues [8–10]. The IMB model of health behavior modification presumes that executing a health promotion behavior is a function of the extent to which one is well informed about the behavior, is motivated to engage in the behavior (e.g., has positive personal attitudes and/or intentions toward the behavior, and social support for the behavior), and has the appropriate behavioral skills to perform the behavior [11,12]. Substantially, one who is knowledgeable and motivated is more inclined to develop and enact the related behavioral skills and more likely to engage in the targeted health behavior [11,12]. The IMB model has been extensively applied to predict positive health behavior for over a decade. The model has been found to be transferable to multiple populations [10] such as truck drivers [13], college students in general [8,14], women in low-income housing [15], intravenous drug users [16], underserved minority youth [17], patients with diabetes [18], preschool children [19], incarcerated juvenile offenders [20], among others. However, no study has been published to date with a focus on rational drug use among outpatients.

In this study, we tested a hypothetical theoretical-based model using constructs from the IMB model enhanced with the key demographic variables of age, gender, level of education and household monthly income. We hypothesized that information (e.g., rational drug use knowledge) and motivation (e.g., personal attitudes and/or intentions towards rational drug use behavior and social support for the behavior) would be significantly associated with behavioral skills. In turn, behavioral skills would mediate the effect of information and motivation on rational drug use behavior. Information and motivation might also directly affect rational drug use behavior. Meanwhile, we also assessed whether any of the background demographic variables had an effect on the behavior directly or if the behavior was mediated through information, motivation or behavioral skills. We anticipate that this study will provide direction for future IMB-based rational drug use behavior intervention.

## Methods

### Sample and procedure

We used the multi-stage cluster random sampling method in the study. In the first stage, three prefecture-level cities, namely Bengbu, Hefei and Tong-ling were randomly selected. In the second stage, we randomly chose a district from each prefecture-level city. In the third stage, we randomly chose a second-level hospital from each district as the investigation scene. Finally, we conducted a one-day survey in each hospital, which resulted in 426 cases from Bengbu, 423 cases from Hefei and 411 Tong-ling. To be eligible for the study, the outpatients had to be 18 or older with a clear state of consciousness (e.g., a state of consciousness that includes normal thinking, sharp response, clear language and normal expression) and a willingness to cooperate (e.g., actively cooperate with the investigators to complete all aspects of the questionnaire). After excluding those who did not meet the criteria, our sample size included 1,260 outpatients, from whom 1,214 usable questionnaires were collected for a response rate of 96.3%.

The Ethics Committee of the First Affiliated Hospital of Anhui Medical University approved the study and permitted us to investigate outpatients once they gave verbal consent. After obtaining permission from the outpatients, trained investigators briefly introduced to the survey and instructed each outpatient to complete the questionnaire in 15 minutes. To protect the privacy of the outpatients, the questionnaire did not include any identifying information. We employed a face- to- face inquiry method during the investigation with the investigator reading each item to the participant and recording the participant’s response. The survey was conducted from July 3, 2014 through July 9, 2014.

### Measures

We systematically summarized relevant background research and further redesigned the questionnaire based on the literature review. With respect to the structure of the questionnaire, we reviewed and adopted the established methods of questionnaires regarding HIV/AIDS [8,21] and tuberculosis (TB) prevention behavior intervention research [22]. With respect to the content of the questionnaire, we mainly referred to *The Core Information Interpretation of Health Education in Rational Drug Use* [2]. The original questionnaire was developed using the expert consultation method, and it was improved by examining participants' understanding of the questionnaire items and the content validity of the questionnaire after implementing the preliminary investigation.

The questionnaire consisted of five parts: demographics, rational drug use information, rational drug use motivation (e.g., personal motivation and social motivation), rational drug use behavioral skills and rational drug use behavior. The content of the questionnaire was intended to cover all aspects of the rational use of drugs. Therefore, the scores on four dimensions, namely information, motivation, behavioral skills and behavior, were incorporated into the model test. Subsequently, the demographic variables were included as background predictors, and the four summed scales (information, motivation, behavioral skills and behavior) were analyzed using a structural equation model (SEM) with a measured approach for further analysis to represent the four rational drug use constructs.

### Demographics

Demographic information included age in years, gender (male = 1, female = 2), level of education (primary school or below = 1, junior high school = 2, senior high school/technical secondary school = 3, junior college = 4 and bachelor’s degree or above = 5) and household monthly income (<2,000 Chinese yuan = 1, 2,000 to 3,999 Chinese yuan = 2, 4,000 to 5,999 Chinese yuan = 3, 6,000 to 7,999 Chinese yuan = 4, and ≥8,000 Chinese yuan = 5).

### Information

Twenty informational type items (Cronbach’s alpha = .782) required a "yes" or "no" response. A participant's score on the information subscale was based on the total number of correct responses out of 20. The typical items from the scale included, "Rational drug use is referring to safe, effective and economical drug use"; "Pregnant women, children and the elderly can be guided to medicate according to the general adult standard"; and "The reimbursement ratio of essential drugs is higher than the non-essential drugs."

### Motivation

The motivation subscale was assessed using 22 items (Cronbach's alpha = .717), included two parts: personal motivation and social motivation (social support). Typical items from the personal motivation scale included, "Will you ignore the rational use of drugs while focusing on the drug price?"; "In the process of doctors' prescriptions, do you have preference for using antibiotics?"; and "Are you willing to further study and understand the knowledge about the rational use of drugs?". Typical items from the social motivation scale included "Does the family support your rational drug use behavior?" and "Is media advertising helpful for you to choose some safe, economic and effective drugs?". The response options were divided into four categories ranging from 1 (completely yes) to 5 (completely no), 1 (always) to 5 (never), 1 (very willing) to 5 (never) or 1(very supportive) to 5(never supportive). The items were scored from 1 to 5 or reverse-scored. Therefore, higher scale scores indicated more motivation.

### Behavioral skills

The behavioral skills scale score was based upon each outpatient’s responses to 2 items (Cronbach's alpha = .923): (1) "If there are some education and publicity activities related to rational use of drugs or essential drugs, would you like to participate in?”; and (2) "Would you like to communicate with friends and family about your knowledge of rational drug use and your feeling of the use of essential drugs?" Response options ranged from 1 (very willing) to 5 (never). The item scores were reverse-coded before the scale scores were computed. Higher scale scores reflected higher levels of behavioral skills.

### Behavior

The behavior subscale was indicated by 7 items. Typical items from the scale included, "For the daily medication, do you medicate in accordance with the drug instructions?”; "Do you strictly follow the doctor's prescriptions regarding the use of drugs?"; and " For the daily purchase of drugs, do you go to the official medical institutions or pharmacies to buy drugs?”. Response options ranged from 1 (always) to 5 (never). The items were reverse-coded before the scale scores were calculated. Higher scale scores indicated more rational drug use behavior.

### Statistical analysis

The ‘Complex Samples Procedure’ of the Statistical Package for Social Science (SPSS version 17.0) was used to perform the statistical analyses. The hypothetical SEM was estimated using AMOS 7.0.

A bivariate analysis provided the relationships among the demographic variables and the model variables. Subsequently, the IMB model and structural equation modeling (SEM) were used in this study to examine relations among the demographic variables and the model variables. According to the assumptions and the bivariate analysis, we constructed the original pathways of the hypothesized structural equation model (SEM). Initially, pathways with no significance in the hypothesized model were included as verification of no relationship. However, in the end, non-significant pathways and associations were gradually eliminated until only significant pathways remained.

The N:q ratio (where q represents the number of estimated free parameters) is a commonly-accepted power indicator in SEM [23]. With an approximate N:q ratio of 45:1, our sample (N = 1,214) was sufficient to test our proposed model. According to Kline [24] and Garson’s [25] interpretation of model fit, the likelihood ratio by the chi-square, the comparative fit index (CFI) and root mean square error of approximation (RMSEA) were calculated. A structural model has good data fit if the chi-square is non-significant (>.05), CFI approaches 1.0 and RMSEA is≤.05.

## Results

### Sample characteristics

Outpatients were, on average, 45.3 years old (Table 1). Females made up 64.4% of all outpatients. Level of education was stratified with 10.4% having gained bachelor’s degree or above, 16.2% having completed junior college, 23.6% having completed high school or technical secondary school and approximately 49.8% not completing high school. Household monthly income was varied with 6.7% reporting an income of <2,000 Chinese yuan (1USD = 6.21 CNY), 48.4% reporting an income of 2,000 to 3,999 Chinese yuan, 30.3% reporting an income of 4,000 to 5,999 Chinese yuan, 10.1% reporting an income of 6,000 to 7,999 Chinese yuan and the remaining 4.5% reporting an income of ≥8,000 Chinese yuan.

**Table 1:** Participant characteristics using the Complex Samples Procedure (N = 1,214).

Characteristic variables	N (%)
**Age (in years)**	45.3±16.7
≤30	300(24.7)
31~45	344(28.8)
46~60	306(25.2)
≥61	264(21.7)
**Gender**	
Male	432(35.6)
Female	782(64.4)
**Level of Education**	
Primary school or below	246(20.3)
Junior high school	359(29.6)
Senior high school /Technical secondary school	286(23.6)
Junior college	197(16.2)
Bachelor’s degree or above	126(10.4)
**Household monthly income (CNY** * **)**	
<2,000	81(6.7)
2,000~3,999	587(48.4)
4,000~5,999	368(30.3)
6,000~7,999	123(10.1)
≥8,000	55(4.5)

*.*CNY* Chinese yuan; 6.21 CNY = 1 USD

### Bivariate analysis

(Table 2) presents the associations among the demographic variables and constructs in the IMB model. Age only exerted minor influences in the bivariate analysis. Older outpatients had less knowledge (-.29, p < .01), less motivation (-.09, p < .05) and less behavioral skills (-.06, p < .05). Compared with males, females had more motivation (.10, p < .01), more behavioral skills (.12, p < .01) and more behavior (.10, p < .01). Higher-education outpatients had more knowledge (.55, p < .01), more motivation (.22, p < .01), more behavioral skills (.22, p < .01) and more behavior (.27, p < .01). Higher-income outpatients reported greater knowledge (.18, p < .01), more behavioral skills (.09, p < .01) and more behavior (.10, p < .01). Knowledge, motivation and behavioral skills were significantly interrelated and were all positively associated with behavior.

**Table 2:** Correlations among demographic variables and constructs in the IMB model using the Complex Samples Procedure.

Variables	1	2	3	4	5	6	7	8
**1.Age**	__							
**2.Gender**	-.06*	__						
**3. Education**	-.41**	-.02	__					
**4.Income**	-.12**	-.03	.24**	__				
**5.IMB Information**	-.29**	.05	.55**	.18**	__			
**6.IMB Motivation**	-.09*	.10**	.22**	.04	.27**	__		
**7.IMB Skills**	-.06*	.12**	.22**	.09**	.24**	.49**	__	
**8.Rational Drug Use Behavior**	-.05	.10**	.27**	.10**	.38**	.55**	.43**	__

*p<.05;

**p<.01

### Path analysis

**Fig 1:**
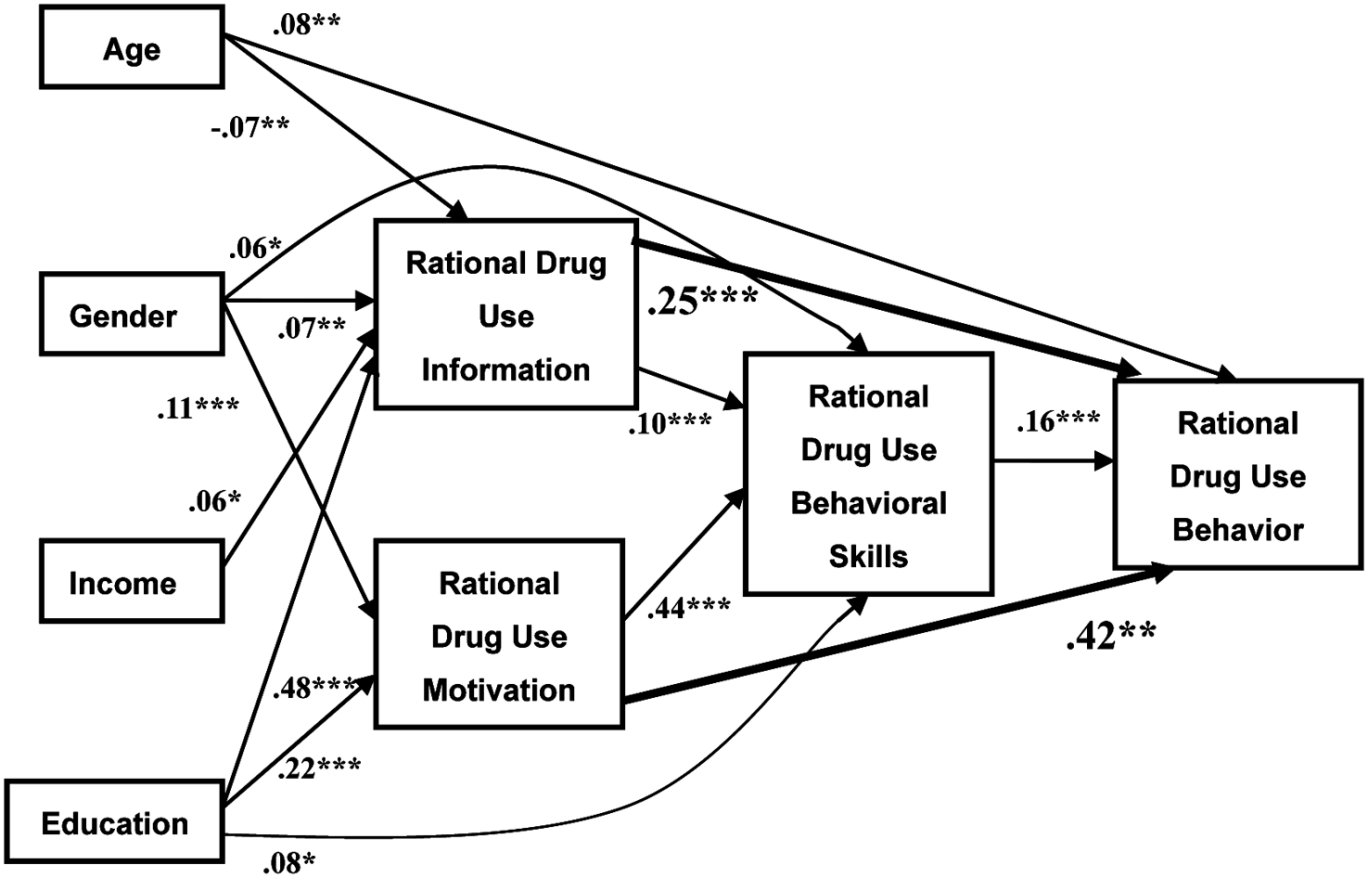
Structural equation model depicting significant regression paths in the IMB model for outpatients (N = 1,214). Single-headed arrow indicates regression path. Regression coefficients are standardized (*p< .05; **p< .01; ***p < .001).

The hypothesized IMB model with parameters and tests of significance of individual paths is presented in Fig 1. The hypothesized model demonstrated a good fit to the data, (ML χ^2^ (9) = 12.799, p = .172, CFI = .998, RMSEA = .019; 90% CI: .00-.04). Moreover the IMB model predicted 40% of the variance for behavior and 27% of the variance for behavioral skills.

#### Direct effects

Rational drug use behavior was predicted by better behavioral skills (standardized regression coefficient = .16, p < .001), older age (.08, p < .01), greater knowledge (.25, p < .001) and more motivation (.42, p < .001). Behavioral skills were significantly predicted by female gender, higher educational level (.08, p < .05), greater knowledge (.10, p < .001) and more motivation (.44, p < .001). Older outpatients reported less knowledge (-.07, p < .01). Compared with males, females had greater knowledge (.07, p < .01) and more motivation (.11, p < .001). Higher-income outpatients reported more knowledge (.06, p < .05). Outpatients with higher educational levels had greater knowledge(.48, p < .001)and more motivation (.22, p < .001) (Fig 1).

Significant *indirect effects* on behavior included greater knowledge (.02, p < .05), more motivation (.07, p < .05), age (p < .05), gender (p < .05), household monthly income (p < .05), level of education (p < .05). All demographic variables had significant indirect effects on behavioral skills as well (Fig 1).

## Discussion

The results of the model indicated that information and motivation not only affected behavior indirectly through behavioral skills but also directly influenced behavior. Our data also showed that the direct effect of information on behavior (.25) was greater than the indirect effect of that on behavior (.02) and that the direct effect of motivation on behavior (.42) was greater than its indirect effect on behavior (.07). These results indicated that behavioral skills partially mediated the influence of information and motivation on behavior. The relevant research by Fisher, et al. [26, 27], however, indicated that the impact of information and motivation on behavior was mainly mediated by behavioral skills. There could be three reasons for our results. First, it seemed that the heterogeneity due to different levels of diversity among drug-using outpatients in the sample might be related to the outcomes in our study, in contrast to the works of Fisher, et al. [26,27], whose samples from people at high risk for HIV/AIDS were mostly homogeneous. Second, the IMB model was applied to the rational use of drugs for the first time, and thus, its indicators might not be sufficiently comprehensive but rather in need of further improvement. Finally, there was evidence in the relevant literature [27] to suggest that the direct pathways regarding the effect of information and motivation on behavior did not occur when an anticipated behavior was particularly complex or needed multiple behavioral skills to enact. In the case of the present study, there occurred the direct pathways about the effect of information and motivation on behavior in the model. Therefore, it was likely that rational drug use behavior was not complex, that complex or new behavioral skills did not appear in the enforcement of the practice, or that the selected indicators of behavioral skills did not cover all relevant aspects of those skills [27, 28].

On the one hand, the above results of the model corroborated the hypothesis that relationships between information and motivation with rational drug use behavior were at least partially mediated by behavioral skills, while information and motivation exerted a direct effect on rational drug use behavior. Although the direct effect of behavioral skills on behavior was less than that of information and motivation on behavior, a partial response regarding behavioral skills provided a clue for their further improvement and, hence, for rational drug use. Consistent with the IMB model, more information, more motivation and more behavioral skills could increase rational drug use behavior. On the other hand, these findings suggested that an IMB-based intervention would affect rational drug use behavior by affecting these IMB variables. Consequently, in the future, rational drug use behavior interventions for outpatients should not only be confined to knowledge education but also should pay close attention to the important roles of motivation and behavioral skills. At the same time, Anderson's research has demonstrated that the level of motivation and behavioral skills after the intervention could better reflect the changes in behavior and that when just limited to knowledge level indicators, the quality of interventions might not be able to scientifically reflected [15]. Therefore, the IMB model provides a valid theoretical framework for future rational drug use behavior interventions.

Our data showed that age had a significant minor influence on information and behavior. The results suggested that older age corresponded to less rational drug use information and more rational drug use behavior. In this study, outpatients aged 46 and older made up 46.9% of all outpatients, and outpatients aged 61 and older accounted for 21.7%. Therefore, the proportion of elderly outpatients was quite significant in our study. In our investigation, the level of education was varied with approximately 64.0% not completing senior high school and only 36.0% having completed senior high school or above among outpatients aged 46 and older. Therefore, the older subjects had less basic knowledge about the rational use of drugs, which could be related to their lower level of education. Meanwhile, other research has indicated that older patients paid more attention to their health [29], which might make them medicate more cautiously and rationally. Due to their prevailing lower level of education, this population might encounter some difficulties in the process of acquiring and knowing knowledge about the rational use of drugs while they attached more attention to health [29]. Therefore, while this group is given knowledge education, they require outside support for the rational use of drugs as well. Meanwhile, other studies have suggested that social support was critical to executing relevant behavior [30, 31]. If a high level of social support was perceived, personal attitudes toward the behavior would be more positive [32]. Accordingly, we recommend that family members, friends and medical personnel take responsibility for supporting rational drug use in this group of patients.

These data also showed that educational level was strongly associated with information and motivation. The results suggested that outpatients with higher educational levels had greater information and more motivation. With respect to information, perhaps the better educated patients might have a relatively stronger thirst for knowledge as well as more means to acquire knowledge. Meanwhile, studies found that patients’ accumulation of medication knowledge required them to have a certain level of education [3]. Regarding motivation, the individuals with greater education might have a more positive attitude toward rational drug use, and they might also have the ability to highly perceive the risks of irrational drug use and obtain more social support for rational drug use from family members and/or friends.

Although demographic variables such as gender, income and level of education were significantly associated with rational drug use behavior in the bivariate analysis, with the exception of age, they had no direct effect on behavior in the predictive path model. In addition, the data documented a significant relationship between demographic variables and information, motivation and behavioral skills. Consequently, demographic variables exerted indirect impacts on behavior mainly through information, motivation and behavioral skills.

This research indicates that to promote rational drug use behavior among outpatients, the future design of intervention programs for rational drug use should be based on the theory while demographic characteristics should also be taken into account. These findings point to an urgent necessity and a great deal of work needed to design a sound intervention program, at least in China, as far as rational drug use among outpatients is concerned.

There were limitations to this study that should be acknowledged. First, as the IMB model was the first to be introduced to the study of rational drug use for outpatients, the items assessing each part of the IMB model might not be comprehensive. Second, we examined the IMB model using a structural equation model and tested the constructs using cross-sectional data, which could only represent associations between constructs observed at a single point in time. However, the formation of rational drug use behavior is a dynamic process. Only longitudinal research could provide more insight into behavioral problems.

Despite these limitations, our study was the first to utilize the predictive IMB model to evaluate rational drug use behavior among outpatients in China. Furthermore our findings note directions for future IMB-based intervention studies of rational drug use behavior among outpatients.

## Supporting Information

S1 Table Participant characteristics using the Complex Samples Procedure (N = 1,214). *.*CNY* Chinese yuan; 6.21 CNY = 1 USD
(DOC)

S2 Table Correlations among demographic variables and constructs in the IMB model using the Complex Samples Procedure. *p < .05; **p < .01
(DOC)
