# Correction: A Study on the Application of the Information-Motivation-Behavioral Skills (IMB) Model on Rational Drug Use Behavior among Second-Level Hospital Outpatients in Anhui, China

**DOI:** 10.1371/journal.pone.0137928

**Published:** 2015-09-03

**Authors:** Cheng Bian, Shuman Xu, Heng Wang, Niannian Li, Jingya Wu, Yunwu Zhao, Peng Li, Hua Lu

There is an error in affiliation 2 for author Shuman Xu. Affiliation 2 should be: Guangdong Women and Children Hospital, Guangzhou, Guangdong, PR China.

## References

[1] Bian C, Xu S, Wang H, Li N, Wu J, Zhao Y, et al (2015) A Study on the Application of the Information-Motivation-Behavioral Skills (IMB) Model on Rational Drug Use Behavior among Second-Level Hospital Outpatients in Anhui, China. PLoS ONE 10(8): e0135782 doi:10.1371/journal.pone.0135782 26275301 10.1371/journal.pone.0135782 PMC4537188

