# Supplementary material for: A Study on the Application of the Information-Motivation-Behavioral Skills (IMB) Model on Rational Drug Use Behavior among Second-Level Hospital Outpatients in Anhui, China
Source: PLoS One. 2015 Aug 14;10(8):e0135782. doi: 10.1371/journal.pone.0135782 (PMC4537188; doi:10.1371/journal.pone.0135782)
Supplement: S1 Table — *.CNY Chinese yuan; 6.21 CNY = 1 USD (DOC) [file pone.0135782.s001.doc]

**S1 Table. Participant characteristics using the Complex Samples Procedure (N=1,214)**

| **Characteristic variables** | **N (%)** |
| --- | --- |
| **Age (in years)** | 45.3±16.7 |
| ≤30 | 300(24.7) |
| 31~45 | 344(28.8) |
| 46~60 | 306(25.2) |
| ≥61 | 264(21.7) |
| **Gender** |  |
| Male | 432(35.6) |
| Female | 782(64.4) |
| **Level of Education** |  |
| Primary school or below | 246(20.3) |
| Junior high school | 359(29.6) |
| Senior high school /Technical secondary school | 286(23.6) |
| Junior college | 197(16.2) |
| Bachelor’s degree or above | 126(10.4) |
| **Household monthly income (CNY*)** |  |
| ＜2,000 | 81(6.7) |
| 2,000~3,999 | 587(48.4) |
| 4,000~5,999 | 368(30.3) |
| 6,000~7,999 | 123(10.1) |
| ≥8,000 | 55(4.5) |

* .*CNY* Chinese yuan; 6.21 CNY = 1 USD
