# Supplementary material for: A Study on the Application of the Information-Motivation-Behavioral Skills (IMB) Model on Rational Drug Use Behavior among Second-Level Hospital Outpatients in Anhui, China
Source: PLoS One. 2015 Aug 14;10(8):e0135782. doi: 10.1371/journal.pone.0135782 (PMC4537188; doi:10.1371/journal.pone.0135782)
Supplement: S2 Table — *p < .05; **p < .01 (DOC) [file pone.0135782.s002.doc]

**S2 Table. Correlations among demographic variables and constructs in the IMB model using the Complex Samples Procedure**

| **Variables** | **1** | **2** | **3** | **4** | **5** | **6** | **7** | **8** |
| --- | --- | --- | --- | --- | --- | --- | --- | --- |
| **1.Age** |  |  |  |  |  |  |  |  |
| **2.Gender** | -.06^*^ |  |  |  |  |  |  |  |
| **3. Education** | -.41** | -.02 |  |  |  |  |  |  |
| **4.Income** | -.12** | -.03 | .24** |  |  |  |  |  |
| **5.IMB Information** | -.29** | .05 | .55** | .18** |  |  |  |  |
| **6.IMB Motivation** | -.09* | .10** | .22** | .04 | .27** |  |  |  |
| **7.IMB Skills** | -.06* | .12** | .22** | .09** | .24** | .49** |  |  |
| **8.Rational Drug Use Behavior** | -.05 | .10** | .27** | .10** | .38** | .55** | .43** |  |

*p<.05; **p<.01
